# Effectiveness and Feasibility of a Self-guided Mobile App Targeting Emotional Well-being in Healthy Adults: 4-Week Randomized Controlled Trial

**DOI:** 10.2196/44925

**Published:** 2023-04-03

**Authors:** Emily Brindal, Naomi Kakoschke, Sinead Golley, Megan Rebuli, Danielle Baird

**Affiliations:** 1 Commonwealth Scientific and Industrial Research Organisation Adelaide, South Australia Australia

**Keywords:** emotional well-being, self-guided, smartphone app, mobile health, mHealth, affect, mobile phone

## Abstract

**Background:**

Commercial smartphone apps designed to promote emotional well-being are becoming increasingly popular, but few apps have been empirically validated.

**Objective:**

This study examined the feasibility and effectiveness of a self-guided app designed to reduce daily stress via positive messaging and tailored short inspirational talks (ie, peps).

**Methods:**

A total of 166 participants (n=112, 67.5% female; mean age 38.48, SD 6.73 years) were recruited through social media advertising and randomized into an intervention (Hey Lemonade app plus twice daily mood monitoring using the Multidimensional Mood Questionnaire [MDMQ]) or active control (twice daily mood monitoring [MDMQ]) group. Primary (coping self-efficacy [CSE]; 3 subscales) and secondary outcomes (vitality, satisfaction with life, perceived stress, positive and negative affect, and hassles and uplifts) were measured at the baseline (week 1) and end point (week 4). The app evaluation questions were assessed at week 2. All interactions and measurements were collected on the internet and through the apps.

**Results:**

In total, of 166 participants, 125 (75.3%) completed the trial. There were no differences in dropout rates between the groups (62/81, 76% intervention; 63/85, 74% control). There were significant group-by-time interactions for vitality and hassles but no significant effect for CSE total (*P*=.05). For the intervention group, the change from baseline to week 4 was significant for vitality (*P*=.002) and hassles (*P*=.004), CSE total (*P*=.008), and CSE Emotional subscale (*P*=.02). For the control group, any changes over 4 weeks were not significant for any outcome. There was a significant group-by-time interaction for MDMQ calmness (*P*=.04). By week 4, calmness was significantly higher in the intervention group (*P*=.046). Of those in the intervention group at week 2 (n=68), 39 (57%) participants recommended the app and 41 (60%) participants wanted to continue using it. Pep talks and customizable voice options were the most popular features.

**Conclusions:**

Participants who had access to the smartphone app on an as-needed basis over the 4-week trial showed significant improvements in emotional well-being indicators. More broadly, this suggests that simple accessible solutions may generate meaningful well-being outcomes. Whether these changes are sustained and can be generalized to other population groups is yet to be determined.

**Trial Registration:**

Australian and New Zealand Clinical Trials Registry (ANZCTR) 12622001005741; https://www.anzctr.org.au/Trial/Registration/TrialReview.aspx?id=384304&isReview=true

## Introduction

### Background

The COVID-19 pandemic has been linked to measurable decreases in emotional well-being in Australians [1,2], an area of health that is gaining increasing focus worldwide. Although acknowledged as a multidimensional construct, currently, there is no clear consensus on the components of emotional well-being. Emotional well-being often includes mood or affective state, happiness, vitality, and satisfaction with life, with a distinction between psychological and subjective well-being. Subjective well-being captures positive affect and satisfaction with life [3], whereas psychological well-being [4] includes eudemonic aspects, such as purpose, growth, acceptance, and flourishing that align with the self-determination theory [5]. Other terms, such as mental well-being [6] and hedonic well-being, are also used interchangeably with psychological well-being. Although some well-being outcomes are used to capture the presence of mental illness, they are distinct. For current purposes, we use the term emotional well-being to reflect a multidimensional construct that includes a variety of emotional states.

Markers of emotional well-being have important clinical implications, given that they are associated with symptoms of anxiety and depression. There is growing evidence of the connection with other markers of health. For example, in a large sample of older adults in the United Kingdom (n=8780), self-realization (a concept tied to eudemonic well-being) and mood were positively associated with biological markers of inflammation in a model controlling for various confounders, including health conditions and mental health symptoms and conditions [7]. A recent meta-analysis of 17 papers suggested that emotional well-being influences changes in physical health in diseased populations [8]. Recent reviews have also indicated that positive mental well-being facilitates recovery from physical illness including cardiovascular disease [9,10].

Stress is conceptually related to well-being. Stress is a psychological response to a situation that exceeds a person’s resources or the product of a person being unable to achieve their immediate goals [11,12]. Stress can be immediate, acute, or chronic. It is widely accepted that stress has a negative impact on both mental and physical health owing to hormonal changes [13] or oxidative stress [14] triggered by psychological stress, which is often suggested as a possible cause. Although stress is often researched in the context of workplaces, it has also been examined in terms of microstressors or daily hassles. Although these may not feel like significant events or intense chronic challenges such as those associated with occupational stress, daily hassles are also likely to be important predictors of health outcomes [15,16].

One theory that combines the concepts of affect, psychological well-being, and stress is the Conservation of Resource (CoR) theory [17,18]. This model suggests that people have certain resources that they can use to regulate their behavior. Resources include those that are psychological or emotional, physical (ie, tools and resources), and relational (ie, social support). For example, positive affect and higher resilience have been theorized to improve a person’s ability to manage stress. This theory can also accommodate the experience of daily hassles, which, although seemingly small, also dip into a person’s resources. Coping strategies and ability can also become a resource within this framework. Nevertheless, the CoR theory provides a functional descriptive framework with no obvious avenues for intervention.

App-delivered health programs have grown substantially with the popularization of smartphones [19] and have the potential to become physical resources and social support tools within the CoR framework. Apps have recently been developed in the emotional well-being space [20]. A review of apps targeting emotional well-being available in the Apple App Store and Google Play revealed a total of 231 available apps in 2020, which were largely self-guided in nature [21]. Another review [22], conducted in 2021, revealed 4 different mental health apps with associated published scientific articles. Most of these apps delivered a form of therapy that targets depressive symptoms. The authors concluded that more robust research is required in this area.

### This Study

Emotional well-being is an important area, and apps have the potential to play a role in enhancing positive mental health through emotional well-being [23]; however, few apps targeting improved well-being have been empirically validated for their effectiveness. Thus, this study aims to examine both the feasibility and effectiveness of a self-guided smartphone app called Hey Lemonade, which was designed to help manage everyday stress and improve resilience and emotional well-being via short, pragmatic, and uplifting talks, which are referred to as peps.

## Methods

### Participants

We aimed to recruit approximately 150 adults aged 25 to 50 years, including 38 (25%) of whom were men. A comparison of 100 completers would allow the detection of a medium to large effect size (0.4) in coping self-efficacy (CSE; with a magnitude of a 0.84 difference based on a previous study [24]).

The selected age range and gender proportion were chosen, as they reflected the target audience of the Hey Lemonade smartphone app. Other inclusion criteria were as follows: owning a device with an operating system suitable for installing the Hey Lemonade app and currently residing in Australia. Exclusion criteria were self-reported and included having a professionally diagnosed mental health disorder, currently experiencing an abnormal level of life stressors (eg, loss of job or death of a loved one), being related to or close friends with the research team or developers of the Hey Lemonade app, and having fewer than 11 apps of any nature installed on their phone. This final criterion was chosen as an indicator of low app engagement and possibly app literacy [25]. People experiencing considerable life stressors or mental health diagnoses were excluded because the app was not designed to support major and chronic well-being issues.

### Design

In this study, we used a 4-week randomized controlled trial design comparing the Hey Lemonade app (intervention plus daily mood monitoring) with an active control group (daily mood monitoring only). Participants were not blinded to the name of the intervention app and were informed that the study would evaluate the effectiveness of an app for managing everyday types of stressors. Those in the control group used only a freely available ecological momentary assessment app (SEMA3) to capture their daily mood data [26] (described further in Table 1). This was considered an active control because regular mood monitoring can itself have mental health benefits [27] and has been included as an aspect of most apps targeting emotional well-being [22].

**Table 1:** Secondary outcomes measured at baseline and end point.

Construct	Measure	Items and scale	Scoring	Cronbach α
Subjective well-being	Satisfaction with Life Scale [28]	5 items rated on a 7-point agreement scale	Scores comprised a summed raw score ranging from 5 to 35, with higher scores representing higher life satisfaction.	.88
Affect	Positive and Negative Affect Schedule [29]	20 items rated over the past month from 1 to 5 for the level of extent experienced. Half of the items measure PA^a^, and the other half measure NA^b^	Scores are summed across respective items for PA and NA to represent the strength of affect. Scores can range from 10 to 50, with higher scores representing higher levels of each affect type.	PA: .89 and NA: .83
Stress	Perceived Stress Scale [30]	14 items rated over past month on a 5-point frequency scale	Positively worded questions were reverse scored. Scores for each item were summed to obtain a total score ranging from 0 to 56. Higher scores indicate higher perceived stress.	.84
Daily hassles and uplifts	Adapted^c^ from hassles and uplifts scale [16]	40 items rated as hassles. The same items are then rated as uplifts using the following scores: not relevant (score=0), none (score=1), somewhat (score=2), quite a bit (score=3), a great deal (score=4)	Total hassles and uplifts were calculated at each time point through summing all responses.	Hassles: .90 and uplifts: .84
Vitality	Subjective vitality scale [31]	7 items rated on a 7-point agreement scale	Item number 2 was reverse scored (“I don’t feel very energetic”). All items were summed to calculate an overall score. Higher scores indicate higher vitality.	.89
Momentary mood	MDMQ^d^ [32]	6 bipolar items (eg, “Tired–Awake”) rated “at this moment” using a slider scale from –5 (very) to +5 (very). Entered twice per day within certain time frames only (7-11 AM or PM).	Items assess 3 basic mood dimensions: energetic arousal (E), valence (V), and calmness (C). The MDMQ was delivered using the freely available SEMA3 app [32]. Participants were prompted to complete twice daily assessments. Data entry was only possible during prespecified times. Data from the 3 negative items were reverse coded to ensure higher scores indicated positive valance, energetic arousal, and calmness.	N/A^e^
App usability	N/A	Open-ended questions asking favorite features of the app, what they would change, and how they felt after listening to peps. Recommendation to family and friends (yes; no; unsure) How long do you wish to continue accessing the app? (Not at all; another week or 2; for 2 weeks to a month; for a few months; don’t want to stop) Select favorite 3 features on a prespecified list of 12 core features	Open-ended responses were grouped thematically and then coded a second time to ensure they fit with the core themes.	N/A

^a^PA: positive affect.

^b^NA: negative affect.

^c^The original 53 items were piloted on a small convenience sample (n=17) and refined to a list of 40 options considered more suitable for a contemporary Australian audience. These details are available from the authors on request.

^d^MDMQ: Multidimensional Mood Questionnaire.

^e^N/A: not applicable.

### Intervention: The Hey Lemonade App

The intervention app was developed by Hey Lemonade without input from the authors [33]. It was not publicly available at the time of the trial.

The app’s core function is to provide 3- to 4-minute pep talks about daily stresses that can be customized through the choice of different voice options (referred to as companions in the app). The pep talks were written by professional writers and clinicians, including 2 psychologists, a clinical psychologist, and a solution-focused coaching specialist, who was also a provisional psychologist. The peps were grouped into the following 8 themes: All the Relationships, Daily Pep, Work Pressure, Pump-ups, Pepping Domesticity, Navigating the Feels, Looking After You, and Big Life Stress. Preliminary user experience and interface research led to the development of 30-40 uniquely written peps recorded using 7 voice options. The pep talks were voiced by public figures in the Australian community, representing a diverse mixture of characteristics. Each pep had at least 3 different voice options available.

Pep talks were developed using core principles, including positive affirmation, humor, universality through specificity, and encouraging breathing techniques, as well as using motivational language theory [34] and a solution-based coaching approach [35-37]. The app was designed to be light touch, as users could interact with it whenever they felt they needed additional motivation or inspiration. Therefore, there were no prescribed doses for this interaction. Pep talks could be selected based on theme, browsed by voice options, or users could search for peps on the required topics. Daily peps were offered, but not a mandatory feature to engage with.

Other key aspects of the app included the ability to gift relevant peps to people not using the app, daily pop-up inspirational written quotes delivered via an app notification, and a weekly email from the Hey Lemonade team. As the app was designed to have new content added monthly, new peps and a new voice option were added to the app at the start of week 2 to replicate this feature within the trial period. For further details about the intervention, refer to the CONSORT (Consolidated Standards of Reporting Trials)–EHEALTH checklist [38] provided in Multimedia Appendix 1.

### Measures

#### Primary Outcomes

##### App Engagement

App engagement levels were measured throughout the trial in the intervention group. Data were collected by the app, deidentified, and supplied to us by Hey Lemonade. Overall use was calculated as a summed interaction score (ie, the number of total interactions). This score captures each time a participant engaged with the app in any form. Overall length of any interaction with the app (out of a possible 29 days) was calculated as the difference between the first and the last recorded interaction. If a person had any interaction on day 1 and another on day 28, the length of the interaction was 27 days. This outcome is used to represent an active membership period. Within the app, once a pep had played to completion, users could also rate how they felt: 1=a lot worse; 2=a little worse; 3=about the same; 4=a little better; and 5=a lot better.

##### Coping Self-efficacy

CSE was measured using the 26-item CSE Scale [24] at week 1 (baseline) and week 4 (end point). Participants were asked, “When things aren’t going well for you, or when you’re having problems, how confident or certain are you that you can do the following?” For each item, they were asked to rate the extent to which they believed they could perform behaviors important for adaptive coping on an 11-point scale. Anchor points included 0 (*cannot do at all*), 5 (*moderately certain can do*), and 10 (*certain can do*). Scores were summed for a total CSE score (Cronbach α=.95), as well as for specific domains. The subscales showed excellent consistency: CSE Problem-Focus (12 items; Cronbach α=.89), CSE Stop Unpleasant Emotions (9 items; Cronbach α=.89), and CSE Get Support (5 items; Cronbach α=.85).

#### Secondary Outcomes

Constructs captured as secondary outcomes are described in Table 1. All secondary outcomes were measured at baseline and end point, except for momentary mood, which was captured twice daily, and the app evaluation items measured in the intervention group at week 2.

### Ethics Approval

The study was approved by the Commonwealth Scientific and Industrial Research Organisation Human Research Ethics Committee (application 2022_037_LR) and registered at the Australian and New Zealand Clinical Trials Registry (12622001005741).

### Procedure

Participants were recruited through paid advertisements of Commonwealth Scientific and Industrial Research Organisation on Facebook, targeting people aged 25 to 50 years in October 2022. The Hey Lemonade company directors also promoted the trial on their social media pages (ie, Facebook, Instagram, and Twitter).

Interested participants were directed to a web-based participant information sheet and consent form, which included screening questions to assess eligibility. Those who passed the screening questionnaire were asked to contact the trial manager to express interest in the study. Eligible participants were contacted and asked to provide verbal consent to participate in the study and demographic information. The participants were then emailed the web-based baseline questionnaire. Participants who completed this step were enrolled in the study, given a participant identifier, randomized via a computer-generated sequence on a 1:1 basis ensuring balanced distribution of gender, and provided with instructions about how to install the SEMA3 and Hey Lemonade (intervention-only) apps. The intervention app was installed from the App Store via TestFlight or Google Play using beta testing links because it was not publicly available at the time of the trial. The trial manager randomized the participants, and the research team was blinded to the participant allocation and did not have contact with the participants throughout the study.

Trial outcomes were assessed at baseline, week 2, and end point (week 4). The study was conducted without face-to-face contact. All assessments were completed on the web through the web-based survey platform Alchemer via a link sent to the participants at each assessment point. Participants were sent 1 email reminder if they had not completed the survey within 24 to 48 hours. If they failed to complete the survey after this reminder, they were marked as lost to follow-up. Participants were also able to formally withdraw at any point after the baseline. These participants were asked a reason for their withdrawal. Those who completed the final survey were sent an Aus $50 (US $33.50) electronic gift voucher to thank them for their participation. No costs were associated with accessing any of the apps used in this study.

### Technical Issues

For the Hey Lemonade app, users reported issues accessing the content on 6 occasions. These issues were usually resolved within 24 hours. For the SEMA3 app, approximately 30 participants reported technical difficulties that affected a higher number of Android users. After 3 to 4 days, these issues could not be resolved, so the trial team created a replacement recurring web-based survey to assess momentary mood using the Alchemer platform, in which participants were asked to complete without reminders.

### Data Analysis

To compare the effect of group on change over time for all trial outcome measures, we conducted linear mixed models fitting a marginal model (in which random participant effects are not considered) with unstructured covariance matrices to optimize efficiency [39,40]. Fixed effects included the main effect for group, time, and the interaction effect for group by time, as well as sex. All models were controlled for age as a covariate. The outcomes were analyzed at the intention-to-treat level. Specifically, we used a data augmentation method to analyze the full, incomplete data set, namely the restricted maximum likelihood estimation method [41]. This method does not involve imputing any data but instead uses each participant’s available data to derive maximum likelihood estimates, which refers to the value of the parameter that has the highest likelihood of producing the observed data [42]. The models included all available data from each time point measurement of the 166 participants who commenced the study.

The daily mood data comprised a total of 5305 observations with an average of 31.77 (SD 17.48; range 1-59) observations per participant. There was no significant group difference in the number of observations (intervention: mean 32.92, SD 16.88 vs control: mean 30.64, SD 18.07; t_165_=0.851; *P*=.40). Owing to the technical issues with the SEMA3 app, the number of missing observations in the first week of the trial was high (570/2338, 24.38%), although this was comparable with previous work [32]. Observations for each participant were aggregated by trial week, and within-participant averages from weeks 1 and 4 were compared in subsequent analyses [43]. An unstructured, linear mixed effects model with main effects of group, time, and the interaction effect for group by time was conducted.

SPSS (version 26.0; IBM Corp) for Microsoft Windows was used to perform all analyses, and statistical tests were 2 tailed, with *P*<.05 considered statistically significant. Pairwise comparisons were conducted for all the significant interaction effects.

## Results

### Sample Characteristics

**Figure 1:**
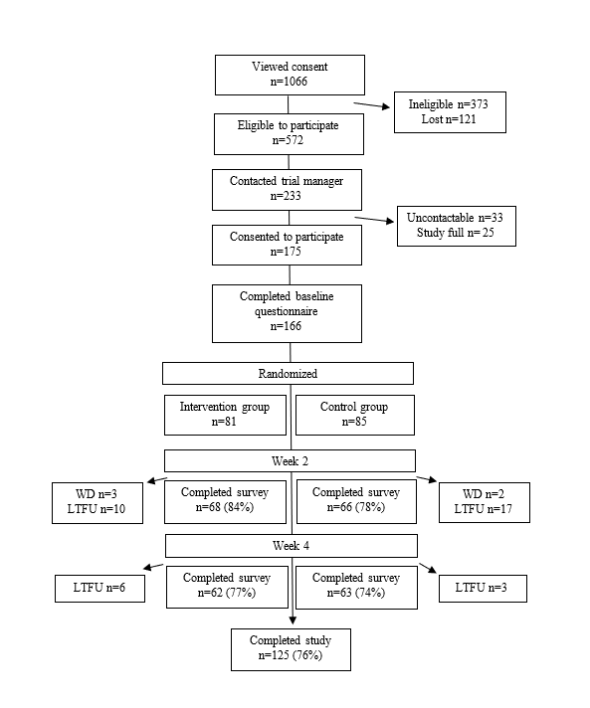
Participant flow diagram. LTFU: lost to follow-up; WD: withdrawn.

A total of 1066 people accessed consent and screening information, with 572 (53.66%) meeting the eligibility criteria. After being contacted by the trial manager, 30.6% (175/572) of individuals consented to participate in the trial. Of the participants who consented 94.9% (166/175) completed the baseline questionnaire and were randomized into either the intervention (81/166, 48.8%) or control (85/166, 51.2%) condition (Figure 1; Table 2). There were no significant group differences in any sample characteristics.

**Table 2:** Sample characteristics^a^ (N=166).

		Intervention (n=81)	Control (n=85)
Female, n (%)		55 (68)	58 (68)
Age (years), mean (SD)		37.63 (6.92)	39.28 (6.48)
**Education, n (%)**			
	High school	5 (6)	5 (6)
	TAFE^b^, trade or certificate	4 (5)	6 (7)
	Diploma	3 (4)	3 (4)
	University degree	35 (43)	29 (34)
	Postgraduate study	34 (42)	42 (49)
**System or device, n (%)**			
	Apple	56 (69)	54 (64)
	Android	25 (31)	31 (37)
**Number of wellness apps installed on phone, n (%)**			
	None	14 (17)	18 (21)
	1-3	42 (52)	38 (45)
	4-9	19 (24)	34 (40)
	10-15	4 (5)	2 (2)
	>15	2 (3)	3 (4)

^a^No significant group differences for any sample characteristics.

^b^TAFE: technical and further education.

A total of 41 participants were lost to follow-up or withdrew from the trial following the completion of the baseline questionnaire and group randomization. There were no significant differences between completers and noncompleters in any of the measures, including demographic variables or baseline primary and secondary outcomes.

There were significant, moderate positive correlations between the primary outcome measure, total CSE, with positive affect (*r*=0.51; *P*<.001), as well as life satisfaction (*r*=0.47; *P*<.001) and vitality (*r*=0.59; *P*<.001). Moderate negative relationships were found between CSE and negative affect (*r*=−0.51; *P*<.001), hassles (*r*=−0.44; *P*<.001), and perceived stress (*r*=−0.61; *P*<.001). Uplifts were weakly to moderately positively associated with CSE (*r*=0.28, *P*<.001).

### Changes in Primary Outcomes

#### App Engagement

The number of users accessing the app each day fell the most dramatically during the first week and continued to taper off for the remainder of the trial. When interactions were divided by the number of active users per day, the intensity of interaction per user remained stable from day 5 (Table 3). Most participants in the intervention condition accessed the app on the first day (68/81, 84%) or the second day (10/81, 12%) it was available. Overall, app membership (the mean number of days between the first and the last interaction) was 19.15 (SD 8.40; range 0-29) days. One participant did not access the app until 18 days after it became available. A total of 3 users who failed to complete the study outcomes at week 2 had memberships from 19 to 29 days.

**Table 3:** Total interactions, number of users, and interactions divided by number of users over the 4-week study period.

Date	Total interactions, n	Number of users, n	Interactions/users
August 18, 2022	3401	69	49.29
August 19, 2022	2351	68	34.57
August 20, 2022	969	52	18.63
August 21, 2022	1469	46	31.93
August 22, 2022	941	47	20.02
August 23, 2022	710	36	19.72
August 24, 2022	651	33	19.73
August 25, 2022	377	31	12.16
August 26, 2022	211	23	9.17
August 27, 2022	310	21	14.76
August 28, 2022	375	26	14.42
August 29, 2022	335	27	12.41
August 30, 2022	293	20	14.65
August 31, 2022	248	18	13.78
September 1, 2022	314	21	14.95
September 2, 2022	215	16	13.44
September 3, 2022	129	11	11.73
September 4, 2022	374	16	23.38
September 5, 2022	302	21	14.38
September 6, 2022	271	20	13.55
September 7, 2022	238	12	19.83
September 8, 2022	184	10	18.4
September 9, 2022	178	12	14.83
September 10, 2022	113	7	16.14
September 11, 2022	94	7	13.43
September 12, 2022	105	9	11.67
September 13, 2022	167	7	23.86
September 14, 2022	188	10	18.8
September 15, 2022	323	12	26.92
September 16, 2022	165	15	11

The mean intensity of app use over the intervention period (summed interaction score) for those who completed the intervention (n=62) was 213 (range 9-936). One-third of the users who completed the study had between 100 and 200 interactions with the app, whereas 1 *superuser* had 936 interactions.

In the multiple regression analysis, participant sex, age, number of wellness apps, and device type failed to predict higher total engagement with the app over the trial, explaining 2.7% of the total variance (*F*_4,76_=1.55; *P*=.20).

The frequency of pep selection per participant was used as the primary engagement metric. There was a total of 36 different pep talks available for selection, which were played on 914 individual occasions. Of the 81 participants randomized to the intervention app, 80 accessed at least 1 pep. Users accessed peps between 0 and 74 times each throughout the trial, with roughly one-third accessing a pep 1 to 4 times, 5 to 11 times, or 12 to 48 times, and a single user accessing a pep 74 times. On average, all users in the study listened to a pep 11 (mean 11.65, SD 12.69) times per month. Of all peps accessed, 66.2% (605/914) were marked as finished. A total of 14% (11/81) of users failed to play a pep to marked completion at any point throughout the trial.

Once a pep had been played to completion, participants were offered the opportunity to indicate the extent to which listening to the pep had impacted how they were feeling, a feature included for the trial duration only. Of all the peps marked to completion, 81.5% (493/605) received feelings ratings. On the basis of individual pep topics, more than half of the (20/36, 56%) available topics were rated ≥4 on average (“a little better” to “a lot better”), with the mean of all ratings being 4.01 (SD 0.71). On average, each pep was rated ≥3 (“about the same”). The 2 most popular peps were accessed 133 and 128 times throughout the trial, with the third to fifth most popular accessed 47 to 41 times. A total of 9 peps were accessed less than 10 times throughout the trial.

#### Coping Self-efficacy

**Figure 2:**
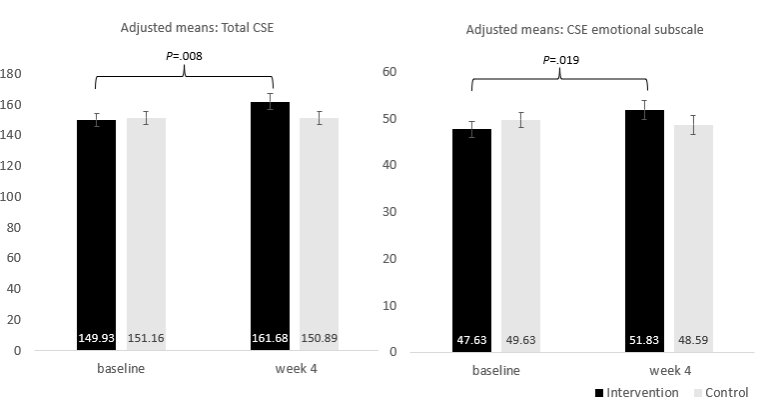
Adjusted means for interaction between group and time for coping self-efficacy (CSE).

The raw means are presented in Table 4, and the linear mixed model results are presented in Table 5. There was a borderline effect for CSE total (*P*=.05). Exploratory analyses were conducted, and models were created using the 3 subscales of CSE to understand if these changes occurred in specific domains. The results revealed a significant interaction effect only for the CSE Emotional subscale (*P*=.04). The only significant pairwise comparison was for the intervention group, with a significant improvement in the CSE Emotional subscale from baseline to end point (*P*=.02; Figure 2).

**Table 4:** Raw means and SDs for outcome measures at baseline and end point presented by group.

		Baseline, mean (SD)		End point, mean (SD)	
		Intervention (n=81)	Control (n=85)	Intervention (n=62)	Control (n=63)
**CSE^a^ total**		150.11 (37.53)	152.56 (38.08)	162.92 (37.65)	154.21 (41.60)
	CSE—problem-focused	73.35 (16.03)	74.87 (17.97)	79.39 (16.03)	75.94 (18.00)
	CSE—stop unpleasant emotions	47.44 (15.20)	49.96 (14.32)	52.21 (15.51)	49.84 (16.57)
	CSE—get support	29.32 (10.27)	27.73 (9.60)	31.32 (9.92)	28.43 (10.34)
Vitality		27.40 (7.49)	27.80 (7.63)	30.21 (7.41)	27.76 (9.03)
Positive affect		31.49 (7.24)	31.56 (7.20)	32.73 (6.83)	31.22 (7.64)
Negative affect		18.64 (5.65)	19.09 (5.71)	19.44 (6.20)	20.67 (6.98)
Satisfaction with life		24.36 (5.86)	24.02 (5.93)	25.98 (6.02)	24.10 (7.44)
Perceived stress		23.77 (6.50)	24.19 (7.01)	24.35 (5.95)	25.32 (7.99)
Hassles		61.57 (14.18)	62.08 (15.11)	56.82 (11.98)	62.14 (14.90)
Uplifts		67.85 (16.42)	67.72 (14.40)	68.71 (15.05)	65.56 (15.70)
**Momentary mood (MDMQ^b,c^)**					
	MDMQ—calmness domain	1.37 (1.55)	1.26 (1.32)	1.60 (1.59)	1.23 (1.43)
	MDMQ—valence domain	1.68 (1.64)	1.75 (1.39)	1.81 (1.80)	1.80 (1.52)
	MDMQ—energetic arousal domain	−0.01 (1.61)	−0.22 (1.34)	0.13 (1.65)	0.00 (1.57)

^a^CSE: coping self-efficacy.

^b^MDMQ: Multidimensional Mood Questionnaire.

^c^On the basis of 1768 observations at baseline and 1130 observations at end point.

**Table 5:** Linear mixed model estimates for primary and secondary trial outcomes.

Model estimates			Fixed effects, B (SE)					
			Intercept	Time (baseline)	Group (intervention)	Sex (female)	Age	Group × time
**Primary outcomes**								
	CSE^a^ total		120.69 (16.91^b^)	0.27 (4.31)	10.79 (6.95)	4.16 (5.99^c^)	0.73 (0.42)	−12.02 (6.12^d^)
	CSE—problem-focused		65.45 (7.39^b^)	−0.63 (2.02)	4.06 (2.99)	1.51 (2.62)	0.23 (0.18)	−5.20 (2.87)
	CSE—stop unpleasant emotions		36.29 (6.65^b^)	1.03 (1.75)	3.23 (2.81)	0.34 (2.335)	0.32 (0.16)	−5.23 (2.49^c^)
	CSE—get support		20.01 (4.42^b^)	−0.20 (1.07)	3.43 (1.77)	2.27 (1.57)	0.16 (0.11)	−1.56 (1.52)
**Secondary outcomes**								
	Vitality		20.11 (3.44^b^)	−0.07 (0.74)	2.20 (1.35)	0.09 (1.22)	0.20 (0.09^c^)	−2.28 (1.05^c^)
	Positive affect		20.93 (3.06^b^)	0.07 (0.82)	1.62 (1.22)	−1.46 (1.09)	0.29 (0.08^b^)	−1.21 (1.16)
	Negative affect		25.80 (2.65^b^)	−1.69 (0.68^c^)	−1.15 (1.16)	−0.52 (0.93)	−0.12 (0.07)	0.50 (0.97)
	Satisfaction with life		19.53 (2.81^b^)	−0.09 (0.55)	1.33 (1.16)	0.69 (0.99)	0.10 (0.07)	−0.82 (0.78)
	Perceived stress		30.06 (3.05^b^)	−0.96 (0.70)	−0.92 (1.19)	−0.92 (1.19)	−0.12 (0.08)	0.30 (0.99)
	Hassles		67.33 (6.34^b^)	−0.66 (1.51)	−5.84 (2.38^c^)	0.20 (2.26)	−0.12 (0.16)	5.12 (2.15^c^)
	Uplifts		51.15 (6.47^b^)	2.03 (1.93)	3.39 (2.65)	0.55 (2.29)	0.36 (0.16^c^)	−2.66 (2.75)
	**Momentary mood (MDMQ^e,f^)**							
		MDMQ—calmness domain	1.06 (0.19^b^)	0.20 (0.16)	0.54 (0.27^c^)	N/A^g^	N/A	−0.05 (0.22^c^)
		MDMQ—valence domain	1.64 (0.21^b^)	0.12 (0.16)	0.16 (0.29)	N/A	N/A	−0.26 (0.23)
		MDMQ—energetic arousal domain	−0.07 (0.22)	−0.14 (0.20)	0.21 (0.30)	N/A	N/A	−0.02 (0.27)

^a^CSE: coping self-efficacy.

^b^*P*<.01.

^c^*P*<.05.

^d^*P*=.05 (approaching significance).

^e^MDMQ: Multidimensional Mood Questionnaire.

^f^On the basis of 1768 observations at baseline and 1130 observations at the end point.

^g^N/A: not applicable.

### Changes in Secondary Outcomes

#### Vitality, Hassles or Uplifts, Affect, Perceived Stress, and Satisfaction With Life

**Figure 3:**
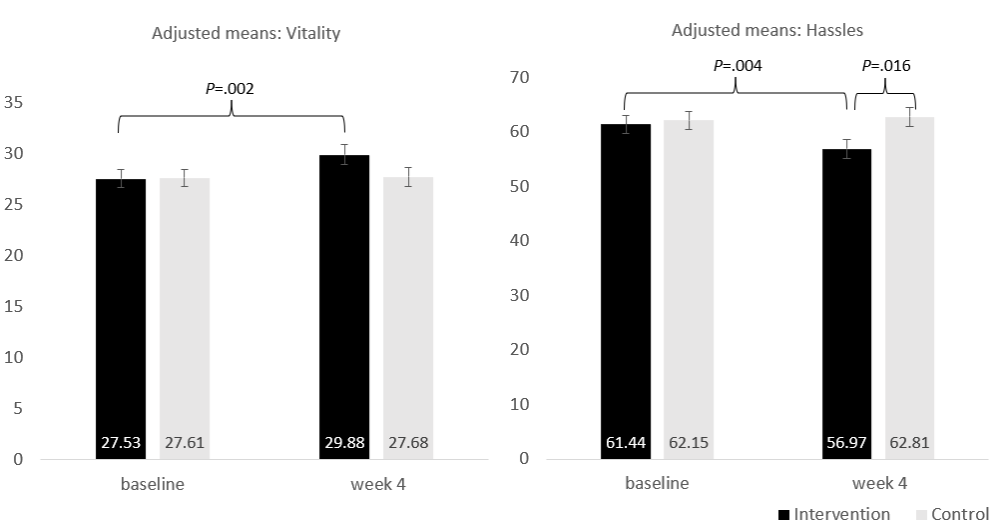
Adjusted means for interaction between group and time for vitality and hassles.

There were significant group-by-time interaction effects for vitality (*P*=.03) and hassles (*P*=.02; Table 4). There were no significant effects for positive and negative affect, perceived stress, uplifts, and satisfaction with life. Pairwise comparisons suggested that at week 4, there was a significant difference between groups for hassles (*P*=.02), with the intervention group reporting lower hassles. The magnitude of the change in the intervention group was significant for vitality (*P*=.002) and hassles (*P*=.004), with the intervention group showing positive improvements in both instances (Figure 3).

#### Momentary Mood

**Figure 4:**
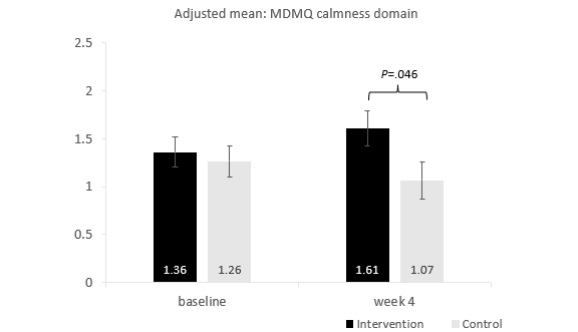
Interaction between group and time for the Multidimensional Mood Questionnaire (MDMQ) calmness domain.

There was a significant interaction between group and time for the momentary mood outcome of the Multidimensional Mood Questionnaire in the calmness domain (*P*=.04). Pairwise comparisons suggested that at baseline, there was no significant group difference in calmness scores (*P*=.67), whereas at week 4, calmness was significantly higher in the intervention group than in the control group (*P*=.046; Figure 4). Pairwise comparisons suggested no significant changes from baseline to week 4 in the intervention (*P*=.10) or control groups (*P*=.21). There were no significant effects for valence or energetic arousal (all *P* values>.10).

#### App Usability

Of the 68 participants in the intervention group at week 2, a total of 39 (57%) indicated that they would recommend the app to family and friends, with a further 16 (24%) who were unsure. A total of 60% (41/68) indicated that they wanted to keep using the app for a few months or indefinitely, with 16% (11/68) not wanting to access the app any further. Pep talks and customizable voice options were selected as the favorite features of the app for most users (Table 6).

**Table 6:** Top features selected by users of the intervention app at week 2 (n=68).

Feature selected^a^	Values, n (%)
The Pep talks	53 (78)
Being able to select different voice companions	43 (63)
The variety of pep talks available	36 (53)
The look and visuals of the app	32 (47)
The tone and style of voices	30 (44)
The material covered by the pep talk	30 (44)
The variety of voice options available	28 (41)
The daily notifications or inspirational quotes	26 (38)
The layout of the menu	19 (28)
The ability to gift a pep	14 (21)
The Peppervescent points	5 (7)
The weekly emails	4 (6)

^a^Each user could select up to 3 options.

In terms of qualitative feedback regarding how they felt after listening to a pep, most participants (49/68, 72%) referenced positive energy with a small number of detractors (5/68, 7%; Table 7).

**Table 7:** Summary of qualitative feedback grouped by key themes with example quotes.

Theme		Quote 1	Quote 2	Quote 3
**Positive energy**				
	Calmer or relaxed (n=17)	“I felt a bit more calm and assured and ready to move forward with my day with more confidence”	“Inspired, calm, armed with practical advice to get through tough times”	“Relaxed and supported.”
	Clearer or uplifted (n=13)	“I felt a ‘pep’ in my step after listening to the talks. I really enjoyed being able to have a quick pick me up from a familiar voice.”	“Clear minded. Refreshed”	“Uplifted and a more positive outlook”
	Generally better or supported (n=13)	“Most often I feel better after listening to a pep talk. I find I use them most when I get stuck in a thought or something happens that derails my day.”	“Help me change the way I think about the issue I'm having.”	“A little better”
	Motivated (n=6)	“Motivated and more positive”	“After some peps, I felt a little better and more motivated”	“Some of them made me feel better, more motivated or happier about the day”
**Null or negative**				
	Neutral (n=14)	“Sometimes I felt a little bit better other times I felt in different”	“Fine but it didn’t tell me anything new”	“About the same, really. It was nice, though.”
	Worse (n=5)	“I couldn't get through the whole thing”	“Patronized. It felt like the app equivalent of someone saying “you’ll be fine, you’re great” when you disclose that you struggle with your self image. Well intended, but misses the mark”	“This isn't for me”

## Discussion

### Principal Findings

In this study, we aimed to evaluate the effectiveness and feasibility of a self-guided app targeting emotional well-being relative to active control of regular mood monitoring. The primary feature of the intervention app was to provide pep talks delivered by familiar and customizable voices to boost people’s mood and coping resources. After 4 weeks, there was no significant change in the total CSE. Exploratory analyses suggested a significant improvement in one component, namely, the perceived ability to stop unpleasant thoughts and emotions (CSE Emotional subscale). The total app use was reduced over the 4 weeks and was not predicted by any participant characteristics. Pep talks, which are the central features of the app, were favorably rated by users. Those who entered the data immediately after listening to a pep largely indicated feeling positive changes in their momentary mood state. Of the 3 aspects of momentary mood measured daily, calmness improved significantly for the app users. Finally, there were substantial improvements in perceived daily hassles and vitality for those randomized to the app condition. Overall, the significant changes observed were aligned with qualitative feedback, which highlighted the impacts of the app on feeling calmer and more positive.

There is very little published evidence in the space of well-being apps, despite the hundreds of apps available for download [21]. Most of the 48 well-being apps reviewed in 2021 assessed mental health outcomes (n=19) and included clinical approaches suitable for these targeted outcomes [22]. Medium effect sizes were reported among the 6 that assessed emotional regulation. Almost all the studies evaluating emotional regulation [22] included full mindfulness interventions, which were largely self-guided intensive programs, including audio sessions ranging between 10 and 30 minutes. The self-guided app that we evaluated was designed to fill a unique space in targeting daily emotional well-being and to be much lighter, which was guided by counseling principles rather than formal, structured programs. Instead of delivering formal therapy, it is more likely to provide a buffer to the constant drain on resources that may lead to larger changes in well-being or increases in stress.

More recently, the Positive Activity Model has supported the idea that simple tools can improve well-being [44]. The current findings support the idea that less intensive intervention apps may be effective. CSE was selected as the primary outcome for this study, given its importance for a range of emotional well-being outcomes. Specifically, it has been flagged as an important mediator of how people manage traumatic events with the potential to mitigate the experience of possible distress [45]. CSE has also been flagged as a relevant factor for maintaining health behavior in the Health Action Process Approach model [46], consuming healthier diets [47], and in elite athlete’s performance [48]. In this trial, the findings for CSE were close to being statistically significant overall, with the subscale measuring the ability to control unpleasant thoughts and emotions showing significant improvement over time in the intervention group. Bandura [49] noted that the ability or inability to control and redirect thoughts is a major aspect of anxiety and that the ability to turn them off is critical for good well-being. As a key feature of the app, it appears that pep talks could assist in developing this critical skill.

App usability and user feedback data were promising. More than 75% (53/68) of participants completing evaluation in week 2 reported wanting to continue using the app beyond the trial. Before commencing, participants were blinded to the content of the app and were told only that the study evaluated its effectiveness on daily stressors. A small number of people did not enjoy the app (ie, 5-11, depending on the metric used). Nevertheless, this sample was not specifically interested in wellness apps, as evidenced by the low number of existing wellness apps installed on the participants’ devices. In combination with partial blinding, it was expected that the app would not be to everyone’s taste. However, the overall qualitative feedback was also mostly positive, reflecting positive changes in calmness while also focusing on feeling positive or having an improved outlook. This appears to have translated to positive changes in momentary calmness and vitality but not overall satisfaction with life.

The hassle conceptualization of stress has been called “a minor events approach” [50] and aligns with the targeted outcome of using the app. Effects were present for this domain but not for general perceived stress. Early conceptions of well-being suggested that hassles were a large part of overall well-being [51] and that hassles were better at predicting well-being, mental health, and health status than life events [50,52]. Using a Dynamic Structural Equation Modeling approach on 14-day data from students, Tran et al [53] reported that physical health complaints were predicted by the experience of hassles the day prior. Overall, the ability to significantly reduce hassles could have health benefits and improve overall well-being. Our measure did not capture the centrality of hassles nor ongoing themes and issues [54]. Future studies could aim to improve measurement to determine if changes occurred for substantial hassles or in sheer volume, which would change the interpretation of possible outcomes.

Hassles may also be reduced through increased vitality, given that positive energy has been associated with fewer negative appraisals of personal problems [55]. Vitality is an important outcome, as it includes feelings of aliveness and high positive energy is associated with motivation [55], which reinforces its potential as a buffer against resource depletion. In recent years, there has been a documented reduction in vitality because of coronavirus-related anxieties [56], meaning that improvements in vitality may be even more timely.

The increase in vitality in the intervention group represented a 7.8% improvement on average. A previous study observed that a 5% to 10% decrease in vitality (based on the 36-Item Short Form vitality subscale) was associated with increased disease risk for conditions ranging from depression to angina and osteoarthritis [57]. Thus, the magnitude of the observed change for the intervention group could have meaningful impacts on future health if these effects can be maintained over the 4-week trial duration.

In the context of a broader theoretical model, it appears that a simple tool can have benefits for some aspects of coping. However, the CoR theory needs to consider not only equipping people with tools but also the confidence to use them [58]. Using a pain paradigm, researchers have shown that greater perceptions of control are most beneficial in the presence of confidence [59]. Self-worth is likely to be an important variable. Future interventions positioning apps as resources for mental health and well-being should account for these variables, as they are likely to have significant mediation or moderation effects on outcomes.

### Strengths and Limitations

This study was a controlled, rigorously designed trial, powered appropriately to detect meaningful effects. Previous studies have used anywhere between 12 days and 12 weeks to evaluate similar apps with those of longer durations targeting clinical samples [21,22]. The 4-week period proved enough to detect changes in some well-being indicators but did not provide an indication of how sustained these changes are over time. This is particularly true for outcomes, such as vitality and hassles, which may be more transient than constructs, such as self-efficacy.

Our primary research questions focused on the effectiveness and feasibility of the app rather than mechanisms for change; therefore, we can only use the literature to guide the interpretation of which specific features of the app were associated with the changes observed. Coaching frameworks, humor [60-62], and positive charismatic people [63] may be beneficial. A total of 2 peps were listened to more than the others, and some of the voice options were also more popular. Appropriate variety in topic and delivery could be an important part of success; however, it is unclear where this tipping point lies.

The current sample was targeted toward individuals in the market for whom the app was developed and designed in a process that occurred before this effectiveness trial. The target market included a narrow age range, comprising mostly women with no major mental health challenges. Approximately one-third of the interested participants were deemed ineligible. It may be possible that healthier samples and women have stronger CSE overall, which makes it easier to build upon [64]. However, its effectiveness in other groups remains unknown. The sample also included a higher proportion of people with university degrees or greater in those aged between 25 and 50 years compared with the general population in Australia, of which 38.7% had obtained this level of education [65]. It is possible that recruitment through our institution’s social media pages attracted more highly educated samples because of its reputation as a national science organization in Australia. However, analysis of other data in Australia suggests that well-being indicators may be harder to shift in samples with greater income or education levels because of higher expectations regarding life circumstances [66].

Not all feedback received regarding the Hey Lemonade app was positive. There was a notable proportion of unsure selections (16/68, 24%) regarding the recommendation of the app to others. Qualitative feedback regarding the ability to gift peps to people provided some insights into possible hesitation to recommend with users, suggesting that they needed to feel comfortable enough to forward on “self-help–type” materials. The nature of a mental well-being focus may create challenges for recommending and sharing components.

One of the largest challenges in this study was the technical issues associated with daily mood monitoring using the freely available SEMA3 app. These may have disproportionately affected the control group, for whom mood monitoring was the primary task associated with trial participation, as well as Android users who appeared to report more technical issues. Unfortunately, using a third-party app, such as SEMA3, meant that these issues were outside the control of the research team. It is reassuring that no negative changes were observed in the control group; however, it is unclear if this may have disrupted any possible positive change generated by mood monitoring for those in the control group.

### Conclusions and Implications

The Hey Lemonade app is designed to be a simple, no-fail tool to assist people in managing daily stress. The intervention did not result in a significant change in the primary outcome of CSE, but it did improve momentary calmness and secondary outcomes, including positive feelings and the ability to cope with general life hassles over 4 weeks. This indicates that a digital intervention such as the Hey Lemonade app might be useful for bolstering people’s resource kitty and hence make them more equipped to face daily challenges. More broadly, the findings also suggest that simple solutions may have the ability to generate meaningful outcomes for well-being [44]. These findings were witnessed in a targeted sample, excluding those inexperienced with apps or experiencing substantial mental health or life challenges. Nevertheless, the Hey Lemonade app represents a promising self-guided approach for managing daily life stress and promoting positive well-being states.

## Appendix

Multimedia Appendix 1 CONSORT (Consolidated Standards of Reporting Trials)-EHEALTH checklist (version 1.6.1).

## Abbreviations

CONSORT: Consolidated Standards of Reporting Trials
CoR: Conservation of Resource
CSE: coping self-efficacy

## References

[1] Brindal E, Ryan JC, Kakoschke N, Golley S, Zajac IT, Wiggins B (2022). Individual differences and changes in lifestyle behaviours predict decreased subjective well-being during COVID-19 restrictions in an Australian sample. J Public Health (Oxf).

[2] Stewart-Brown S (1998). Emotional wellbeing and its relation to health. Physical disease may well result from emotional distress. BMJ.

[3] Diener E (1984). Subjective well-being. Psychol Bull.

[4] Ryff CD (2014). Psychological well-being revisited: advances in the science and practice of eudaimonia. Psychother Psychosom.

[5] Ryan RM, Deci EL (2000). Self-determination theory and the facilitation of intrinsic motivation, social development, and well-being. Am Psychol.

[6] Smith L, Jacob L, Yakkundi A, McDermott D, Armstrong NC, Barnett Y, López-Sánchez GF, Martin S, Butler L, Tully MA (2020). Correlates of symptoms of anxiety and depression and mental wellbeing associated with COVID-19: a cross-sectional study of UK-based respondents. Psychiatry Res.

[7] Fancourt D, Steptoe A (2020). The longitudinal relationship between changes in wellbeing and inflammatory markers: are associations independent of depression?. Brain Behav Immun.

[8] Lamers SM, Bolier L, Westerhof GJ, Smit F, Bohlmeijer ET (2012). The impact of emotional well-being on long-term recovery and survival in physical illness: a meta-analysis. J Behav Med.

[9] Diener E, Pressman SD, Hunter J, Delgadillo-Chase D (2017). If, why, and when subjective well-being influences health, and future needed research. Appl Psychol Health Well Being.

[10] Sin NL (2016). The protective role of positive well-being in cardiovascular disease: review of current evidence, mechanisms, and clinical implications. Curr Cardiol Rep.

[11] LePine JA, LePine MA, Jackson CL (2004). Challenge and hindrance stress: relationships with exhaustion, motivation to learn, and learning performance. J Appl Psychol.

[12] Folkman S, Gellman MD, Turner JR (2013). Stress: appraisal and coping. Encyclopedia of Behavioral Medicine.

[13] Lundberg U (2005). Stress hormones in health and illness: the roles of work and gender. Psychoneuroendocrinology.

[14] Salim S (2016). Oxidative stress: a potential link between emotional wellbeing and immune response. Curr Opin Pharmacol.

[15] Kanner AD, Coyne JC, Schaefer C, Lazarus RS (1981). Comparison of two modes of stress measurement: daily hassles and uplifts versus major life events. J Behav Med.

[16] DeLongis A, Folkman S, Lazarus RS (1988). The impact of daily stress on health and mood: psychological and social resources as mediators. J Pers Soc Psychol.

[17] Hobfoll SE (2002). Social and psychological resources and adaptation. Rev Gen Psychol.

[18] Baumeister RF, Heatherton TF (1996). Self-regulation failure: an overview. Psychol Inq.

[19] (2017). IQVIA Institute for Human Data Science Study: Impact of Digital Health Grows as Innovation, Evidence and Adoption of Mobile Health Apps Accelerate. IQVIA.

[20] Rickard N, Arjmand HA, Bakker D, Seabrook E (2016). Development of a mobile phone app to support self-monitoring of emotional well-being: a mental health digital innovation. JMIR Ment Health.

[21] Marshall JM, Dunstan D, Bartik W (2020). Positive psychology mobile applications for increasing happiness and wellbeing - a systematic app store review. R U appy?. Eur J Appl Posit Psychol.

[22] Eisenstadt M, Liverpool S, Infanti E, Ciuvat RM, Carlsson C (2021). Mobile apps that promote emotion regulation, positive mental health, and well-being in the general population: systematic review and meta-analysis. JMIR Ment Health.

[23] Bakker D, Kazantzis N, Rickwood D, Rickard N (2016). Mental health smartphone apps: review and evidence-based recommendations for future developments. JMIR Ment Health.

[24] Chesney MA, Neilands TB, Chambers DB, Taylor JM, Folkman S (2006). A validity and reliability study of the coping self-efficacy scale. Br J Health Psychol.

[25] (2016). How people discover, use, and stay engaged with apps. think with Google.

[26] Koval P, Hinton J, Dozo N, Gleeson J, Alvarez M, Harrison A, Vu D, Susanto R, Jayaputera G, Sinnott R (2019). SEMA3: Smartphone Ecological Momentary Assessment. Version 3.

[27] Beames JR, Kikas K, Werner-Seidler A (2021). Prevention and early intervention of depression in young people: an integrated narrative review of affective awareness and Ecological Momentary Assessment. BMC Psychol.

[28] Diener E, Emmons RA, Larsen RJ, Griffin S (1985). The satisfaction with life scale. J Pers Assess.

[29] Watson D, Clark LA, Tellegen A (1988). Development and validation of brief measures of positive and negative affect: the PANAS scales. J Pers Soc Psychol.

[30] Cohen S, Kamarck T, Mermelstein R (1983). A global measure of perceived stress. J Health Soc Behav.

[31] Bostic TJ, McGartland Rubio D, Hood M (2000). A validation of the subjective vitality scale using structural equation modeling. Soc Indic Res.

[32] Wilhelm P, Schoebi D (2007). Assessing mood in daily life: structural validity, sensitivity to change, and reliability of a short-scale to measure three basic dimensions of mood. Eur J Psychol Assess.

[33] Hey Lemonade.

[34] Mayfield J, Mayfield M (2018). Motivating Language Theory: Effective Leader Talk in the Workplace.

[35] Grant AM (2012). Making positive change: a randomized study comparing solution-focused vs. problem-focused coaching questions. J Syst Ther.

[36] Grant AM, Gerrard B (2020). Comparing problem-focused, solution-focused and combined problem-focused/solution-focused coaching approach: solution-focused coaching questions mitigate the negative impact of dysfunctional attitudes. Coaching.

[37] Grant AM, O'Connor SA (2010). The differential effects of solution‐focused and problem‐focused coaching questions: a pilot study with implications for practice. Ind Commer Train.

[38] Eysenbach G, CONSORT-EHEALTH Group (2011). CONSORT-EHEALTH: improving and standardizing evaluation reports of web-based and mobile health interventions. J Med Internet Res.

[39] West BT, Welch KB, Galecki AT (2015). Linear Mixed Models: A Practical Guide Using Statistical Software. 2nd edition.

[40] Lu K, Mehrotra DV (2010). Specification of covariance structure in longitudinal data analysis for randomized clinical trials. Stat Med.

[41] Mehrotra DV, Li X, Liu J, Lu K (2012). Analysis of longitudinal clinical trials with missing data using multiple imputation in conjunction with robust regression. Biometrics.

[42] Xi W, Pennell ML, Andridge RR, Paskett ED (2018). Comparison of intent-to-treat analysis strategies for pre-post studies with loss to follow-up. Contemp Clin Trials Commun.

[43] Oleson JJ, Jones MA, Jorgensen EJ, Wu YH (2022). Statistical considerations for analyzing Ecological Momentary Assessment data. J Speech Lang Hear Res.

[44] Lyubomirsky S, Layous K (2013). How do simple positive activities increase well-being?. Curr Dir Psychol Sci.

[45] Sumer N, Karanci AN, Berument SK, Gunes H (2005). Personal resources, coping self-efficacy, and quake exposure as predictors of psychological distress following the 1999 earthquake in Turkey. J Trauma Stress.

[46] Schwarzer R, Renner B (2000). Social-cognitive predictors of health behavior: action self-efficacy and coping self-efficacy. Health Psychol.

[47] Matthews JI, Doerr L, Dworatzek PD (2016). University students intend to eat better but lack coping self-efficacy and knowledge of dietary recommendations. J Nutr Educ Behav.

[48] Nicholls AR, Polman R, Levy AR (2010). Coping self-efficacy, pre-competitive anxiety, and subjective performance among athletes. Eur J Sport Sci.

[49] Bandura A (1988). Self-efficacy conception of anxiety. Anxiety Res.

[50] Chamberlain K, Zika S (1990). The minor events approach to stress: support for the use of daily hassles. Br J Psychol.

[51] Zika S, Chamberlain K (1987). Relation of hassles and personality to subjective well-being. J Pers Soc Psychol.

[52] Weinberger M, Hiner SL, Tierney WM (1987). In support of hassles as a measure of stress in predicting health outcomes. J Behav Med.

[53] Tran ST, Grotkowski K, Miller SA, Reed BW, Koven ML, Buscemi J, Greenley RN (2021). Hassles predict physical health complaints in undergraduate students: a dynamic structural equation model analysis of daily diary data. Psychol Health.

[54] Gruen RJ, Folkman S, Lazarus RS (1988). Centrality and individual differences in the meaning of daily hassles. J Pers.

[55] Ryan RM, Frederick C (1997). On energy, personality, and health: subjective vitality as a dynamic reflection of well-being. J Pers.

[56] Arslan G, Yıldırım M, Aytaç M (2022). Subjective vitality and loneliness explain how coronavirus anxiety increases rumination among college students. Death Stud.

[57] Bjorner JB, Wallenstein GV, Martin MC, Lin P, Blaisdell-Gross B, Tak Piech C, Mody SH (2007). Interpreting score differences in the SF-36 Vitality scale: using clinical conditions and functional outcomes to define the minimally important difference. Curr Med Res Opin.

[58] Benight CC, Ironson G, Klebe K, Carver CS, Wynings C, Burnett K, Greenwood D, Baum A, Schneiderman N (1999). Conservation of resources and coping self-efficacy predicting distress following a natural disaster: a causal model analysis where the environment meets the mind. Anxiety Stress Coping.

[59] Litt MD (1988). Self-efficacy and perceived control: cognitive mediators of pain tolerance. J Pers Soc Psychol.

[60] Wellenzohn S, Proyer RT, Ruch W (2016). Humor-based online positive psychology interventions: a randomized placebo-controlled long-term trial. J Posit Psychol.

[61] Wellenzohn S, Proyer RT, Ruch W (2016). How do positive psychology interventions work? A short-term placebo-controlled humor-based study on the role of the time focus. Pers Individ Dif.

[62] Wellenzohn S, Proyer RT, Ruch W (2018). Who benefits from humor-based positive psychology interventions? The moderating effects of personality traits and sense of humor. Front Psychol.

[63] Bono JE, Ilies R (2006). Charisma, positive emotions and mood contagion. Leadersh Q.

[64] Colodro H, Godoy-Izquierdo D, Godoy J (2010). Coping self-efficacy in a community-based sample of women and men from the United Kingdom: the impact of sex and health status. Behav Med.

[65] (2021). Education and training: census. Information on qualifications, educational attendance and type of educational institution. Australian Bureau of Statistics.

[66] Kristoffersen I (2018). Great expectations: education and subjective wellbeing. J Econ Psychol.

